# Should we vaccinate the better seroconverters or the most vulnerable? Game changing insights for COVID-19 vaccine prioritization policies

**DOI:** 10.1186/s43054-021-00086-8

**Published:** 2021-12-02

**Authors:** Antoine AbdelMassih, Meryam El Shershaby, Hanya Gaber, Menna Habib, Nada Gamal, Reem Husseiny, Nada AlShehry, Abeer Amin, Bassant Heikal, Nadine El-Husseiny, Mai Moursi, Habiba-Allah Ismail, Sara Senoussy, Reem ElSharkawy, Hebat Allah AlZayat, Ghada ElMahdy, Hossameldin Moawad, Ali Genena, Ahmed ElKiki, Mary Reda, Mohamed Khalil, Reem Al Ramady, Noran Radwan, Mariam Khaled-Ibn-ElWalid, Hager Amin, Rafeef Hozaien, Aya Kamel, Raghda Fouda

**Affiliations:** 1 Pediatric Cardiology Unit, Pediatrics Department, Faculty of Medicine, Cairo University, Giza, Egypt; 2 Pediatric Cardio-Oncology Clinic, Children Cancer Hospital of Egypt, Cairo, Egypt; 3 Student and Internship Research Program (Research Accessibility Team), Faculty of Medicine, Cairo University, Giza, Egypt; 4 Faculty of Dentistry, Cairo University, Giza, Egypt; 5 Pixagon Graphic Design Agency, Cairo, Egypt; 6 Clinical and Chemical Pathology Department, Faculty of Medicine, Cairo University, Cairo, Egypt; 7 Pediatrics Department, Faculty of Medicine, Cairo University, Giza, Egypt

**Keywords:** Seroconversion, COVID-19 vaccines, Prioritization policy

## Abstract

**Background:**

With the rapid rise in COVID 19 cases incomparable to the number of vaccinations available, there has been a demand to prioritize the older age groups receiving the vaccine as they have more risk of morbidity and mortality and thus better outcome from vaccination.

**Main body:**

Some studies showed a lower seroconversion rate in older group patients; thus, we discuss the necessity to reprioritize vaccinations to younger age groups who have better seroconversion rates, but we may face some ethical dilemma that could hinder our hypothesis. Decreased seroconversion rates in adults are attributable to immuno-senescence which involves a decrease in humoral and cellular-mediated immunity with age. Despite this fact, there remains some ethical dilemma that can hinder widespread vaccination of younger generations, the most important of which is the unknown long-term effects of COVID-19 vaccines due their fast-tracking under the pressure of the pandemic.

**Short conclusion:**

Prioritizing children vaccination against COVID-19 seems an interesting strategy that can help in containing the pandemic. Resolving some ethical dilemma needs to be done before implementing such strategy.

## Background

The COVID-19 pandemic started in February of 2020. Since then, the virus has spread worldwide, reaching more than 200 million confirmed cases of COVID-19 and more than 4 million deaths (as reported by the WHO on August 6, 2021). Most infected children experienced some fever and general fatigue and recovered without any complications. In contrast, older age groups get severely ill with higher rates of poor recovery and deaths. Vaccination is the only hope to return to regular routines, such as school attendance and public gatherings. A total of 3,984,596,440 vaccine doses have been administered by August 5, 2021. The inability to match the increasing demand for vaccinations with their production led to prioritization policies. Vaccine prioritization policies include the highest risk groups most vulnerable to fatality from the virus; this includes old age and those with underlying health conditions [1].

This prioritization policy is debatable. Seroconversion from vaccination in vulnerable groups is lesser than seroconversion in non-vulnerable groups. Wei et al. studied anti-spike IgG levels in different age groups for two vaccines: ChAdOx1 and BNT162b2. In their study, the elder group had lower seroconversion than the younger-aged group. They noted a marked decline of the titers of anti-spike antibodies with aging. Titers from ChadOx1 were 127 ml in 20-year-old patients and declined gradually to 73 ml in patients above the age of 80. The BNT 162b2 antibody response followed the same pattern across age groups, with a titer of 334 mL in 20-year-old recipients and decreasing to 113 in recipients over 80 years of age [2, 3].

Table 1 discusses the COVID-19 vaccines granted emergency use by the WHO (World Health Organization) and the proper timing of measurement of antibody response to them after vaccination; there is no evidence that time interval for antibody testing differs across age groups [4–6].

**Table 1:** Seroconversion testing granted emergency authorization by the WHO (reference in text [4–6])

o	Trade name	Type	Dosage interval	Least interval for antibody testing after vaccination^a^
mRNA-1273	Moderna	mRNA-based	2 doses, 28 days apart	14 days after 2nd dose
**BNT162b2**	Pfizer/BionTech	mRNA-based	2 doses, 21 days apart	7 days after 2nd dose
**Ad26.COV2.S**	Janssen (Johnson&Johnson)	Non-replicating viral vector	1 dose or 2 doses 56 days apart	14 days after 2nd dose
**AZD1222**	Oxford/AstraZeneca	Non-replicating viral vector	2 doses, 28 days apart	14 days after 2nd dose
**BBIBP-CorV (Vero Cells)**	Sinopharm (Beijing)	Inactivated	2 doses with 2–4 weeks interval between	14 days after 2nd dose
**CoronaVac**	Sinovac	Inactivated	2 doses with 2–4 weeks interval between	14 days after 2nd dose

*Ad* adenovirus, *AZ* Astrazeneca, *BBIBP-CorV* Beijing Institute of Biological Products, Sinopharm corona virus vaccine, *BNT* Biontech, *CoV* coronaviridae, *COVID-19* coronavirus disease 2019, *mRNA* messenger ribonucleic acid, *SARS* severe acute respiratory syndrome, *WHO* World Health Organization

^a^Standard antibody test: total immunoglobulin levels to the receptor-binding domain of the SARS-CoV-2 spike protein were measured with an anti–SARS-CoV-2 S enzyme immunoassay

We thereby hypothesize that vaccinating the younger generations, who are more liable to seroconvert, might provide better community protection than the current vaccine prioritization policy. This might raise some ethical considerations, which we will be discussing at the end of this article.

## Main text

### Molecular mechanisms underlying decreased seroconversion rates in old age

Several cellular and molecular changes are associated with aging. Including thymic involution, oxidative stress, proteostasis, telomere attrition, epigenetic alterations, DNA damage signaling, epigenetic alterations, and transcriptional deviations. These changes affect the immune response to vaccination or infection, leading to immuno-senescence or the gradual decrease in immune system efficiency in the elderly—especially the adaptive immune response. Some of the molecular mechanisms that occur as part of immuno-senescence include both cellular and humoral immunity [7].

#### B cells

As part of the aging process, the body produces fewer B cells. In mice, bone marrow inflammation and decreased production of IL-7, used for the proliferation of naive B cells, led to a reduced output of B cells [8]. Moreover, studies proved an inverse relationship between age and plasma levels of B-cell activating factor (BAF) and a proliferation-inducing ligand (APRIL), both of which can reduce B cell survival in the elderly [9].

Aging does not only affect the number of B cells but also affect their function. There is a decrease in the level of modulators of the B-cell development and differentiation [10]. Namely, E2A gene products are necessary for pro–B-cell production in the bone marrow [11]. RAG enzymes and lambda-5 are also crucial for maturation during the pro and pre–B-cell stages [12].

The long-term function of B cells is affected, too. Class switch recombination (CSR) is the process by which an IgM-producing B cell becomes an IgG-producing B cell. CSR is directly affected by activation-induced cytidine deaminase (AID), regulated by transcription factor E47. Poorer quality E47 mRNA has been found in aged B cells resulting in reduced quantities of AID, and defective CSR and somatic hypermutation [13]. There are also peroxisome proliferator-activated receptors (PPARs), nuclear hormone receptors. They prolong B-cell memory and improve the secondary antibody response. They do so by upregulating anti-apoptotic factors and shifting the metabolism of the B cells to pyruvate kinase to increase their metabolic fitness and enhance the action of the cellular supporters of the B cells (specifically dendritic and T regulatory cells). They also upregulate lipoxin B4, which stimulates B cells to secrete antibodies on re-exposure [14]. PPARs are more abundant in children than adults. So, in conclusion, PPARs’ positive effects on seroconversion and vaccination are reduced with aging [7].

#### T cells

As regards to T cells, firstly, we will be discussing the changes observed in thymopoiesis with advancing age and the suggested molecular causes. The cytokine milieu provides normal thymus function. Due to senescence, cytokines’ expression tends to differ. IL-7 levels do not vary significantly with age, which leads us to believe that changes in IL-7 levels have little effect on thymic involution in the elderly [7, 15]. On the other hand, IL-6 levels increase and cause thymus involution and a reduced T-cell number in peripheral blood. Exogenous IL-6 leads to an acute and rapid response [16, 17]. The sensitivity of the thymus gland to cytokines raises thoughts around using interventional therapy to enhance immune responses [7].

One of the theories mentioned that cytokine milieu, especially IL-6 production, is directly affected by the normal increase of adipogenesis (and atrophy of the thymus tissue) commonly seen with age. Thus, caloric restriction was tested to decrease adipose formation within the thymus and lead to slower involution [18].

Other cytokines such as LIF (leukemia inhibitory factor) and hormones such as corticosteroids have been linked to thymic involution as well [15]. TGFβ also plays a direct role in thymic involution [19].

Tristetraprolin is an RNA-binding protein that is also inactivated with age by P38 mitogen-activated protein kinases (p38-MAPK). Tristetraprolin regulates the transcription of proinflammatory cytokines, which explains their dysregulation with age [7].

Lastly, PPARs, which are less expressed in adults, prolong T-cell memory as well. PPARs achieve the latter by increasing the development of T regulatory cells, which stimulate T cell memory, upregulating γδ type of T cells, which can develop long-lasting memory. Thus, shifting the metabolism of T cells to fatty acid oxidation, critical for the longevity of memory cells and antagonizing PDL, which hinders the development of T memory cells.

PPAR agonists could potentially boost the functions of both B memory and T memory cells to enhance seroconversion [14].

Shifting to myelocytes, a study on the evaluation of innate immunity demonstrated that the pathogen recognition receptor, toll-like receptor TLR1 has reduced expression levels with aging [20].

There is also evidence of cross-regulation between oxidative stress, PPARs, and nuclear factor-kB (NFkB). We know that the induction of PPARs decreases with age and that PPARγ downregulates the activation of macrophages [21]. The dysregulation of NFkB, produced by macrophages, may explain the disrupted inflammatory process with aging since the NFkB pathway is considered a prototypical proinflammatory signaling pathway [7].

Immuno-senescence negatively affects the seroconversion status in the elderly. As such, children might be better seroconverters. Prioritizing children in COVID-19 vaccination programs will ensure administration to those who have the most optimal response to them. Offering protection to children and, indirectly, the elderly through herd immunity.

### Evidence of decreased seroconversion rates in chronic conditions from non–COVID-19 vaccines

Seroconversion is also affected by chronic diseases. One of the pathological factors that might have an impact on seroconversion is chronic kidney disease. Da Roza et al. observed the relationship between patients affected with CKD and their seroconversion status after the HBV vaccine. The primary outcome measure was a hepatitis B surface antibody titer of more than 10 IU 3 months after the vaccination schedule. The study population was 165 patients, including 64% men with a mean age of 60, mean serum creatinine level of 3.4 ± 1.5 mg/dL (300 ± 133 μmol/L), and median estimated glomerular filtration rate (GFR) of 20 mL/min (interquartile range, 14 to 20). Patients with the lowest levels of kidney function and those who were older or had diabetes were less likely to seroconvert [22].

### Evidence of decreased seroconversion in older age from non–COVID-19 vaccines and COVID-19 vaccines

As discussed above, due to an immune function decline, elderly subjects do not respond as efficiently as younger individuals. Some studies show no significant decrease in the seroconversion rate; however, most of the literature supports a significant decline. Some vaccines that have shown decreased seroconversion in the elderly are as follows:

#### Influenza

The Centers for Disease Control and Prevention (CDC) estimates 70–90% clinical efficacy of the influenza vaccine in young adults and 17–53% in the elderly depending on the circulating virus [23].

A systematic review and meta-analysis concluded that the seroprotection rate (SPR) and seroconversion rate (SCR) of older adults for A/H1N1 and B/Victoria were lower than those of younger adults, whereas the two age groups had similar antibody responses for A/H3N2 [24].

Moreover, the H5N1 influenza vaccine effectiveness in older adults may be lower than in younger adults [25].

Frailty (a significant geriatric syndrome characterized by diminished physiologic reserve and increased susceptibility to stressors) was associated with significant impairment in the trivalent inactivated vaccine (TIV)-induced strain-specific hemagglutination inhibition (HI) titers and increased rates of influenza-like illness (ILI) and laboratory-confirmed influenza infection [26].

Furthermore, pre- and probiotics have the potential to modulate antiviral defenses and vaccination responses by altering the gut microbiota. A study in 2018 investigated the effect of a novel probiotic, combined with a prebiotic, on the B and T cell responses to seasonal influenza vaccination in young and older subjects. The profiles of B and T cells differed significantly between young and old subjects. Vaccination increased the number of IgA and IgG memory cells and total IgG B cells in young subjects but not in older subjects and did not affect T cell subsets [27].

#### Hepatitis B

There is no extensive data available regarding hepatitis B. However, some studies have shown an association between age (greater than 50) and decreased seroconversion rates. The study was specifically conducted among health care workers [28].

#### Rabies

Two papers specifically compared seroconversion in different age groups after rabies vaccine administration [29, 30]. These were discussed by Leder et al. [31]. In both studies, responses to purified duck embryo vaccine and HDCV were analyzed.

In the first study, researchers compared subjects aged 11–25 years with those over 50 years of age. All patients were on a 6-dose post-exposure schedule. Both age groups had adequate responses and developed protective antibody titers after the fourth dose, but the older subjects’ antibody titer levels were 52% lower than those of the younger group.

The second study involved 60 patients. They were divided into four age groups: 1–20 years, 21–40 years, 41–60 years, and > 60 years. After 2 doses of the HDCV vaccine, antibody titers were significantly lower for each older group successively. The individual mean titers showed a response that was lower across all the older patients.

#### Japanese encephalitis virus

Two groups with mean ages of 24 and 69 years, were compared with regards to their immune response after receiving a primary vaccination against the travel virus JEV. The older group had significantly lower antibody titers post-vaccination. This titer was presumed to be due to a lower percentage of total and naive B cells in the elderly group as opposed to the younger group [32].

#### Meningitis

General studies performed on the adult show the effectiveness of the vaccine, but it is reported that the efficacy in children under 5 years is low and in those under 18 months practically nil [33, 34].

But it is difficult to generalize because studies carried out in young children are less frequent, and some include only a small number of children [35].

The study was conducted on young participants aged between 18 months and 19 years. The vaccine used was the polysaccharide meningococcal AC. A statistically significant linear trend (*p* < 0.0001) between seroconversion rate and age at vaccination (38.2% in children aged 18 ± 24 months, 57.5% in those aged 24 ± 35 months, 63.6% in those aged 36 ± 47 months, and 77% in those aged 48 ± 59 months) was found. Children aged over 36 months showed a higher quantitative increase in antibodies than those under this age (*p* < 0.0001). Immunogenicity has not been demonstrated in children aged under 24 months, suggesting that the production of antibodies against the polysaccharide group C is different to children aged over 24 months. They observed that 1 month after vaccination, the quantitative increase in the BA titer was greater above the age of 36 months. This aspect has to be considered when indicating vaccination in young children.

#### Hepatitis A

Hepatitis A vaccine (formalin-inactivated) is highly immunogenic, with seroconversion after a single dose in 90–99% of children 2–16 years old and adults under 77 kg. There are lower one-dose seroconversion rates with increasing age [36]. In young persons, seroconversion is seen in 80–90% within 2 weeks of the first dose, and 95–100% of vaccinees have had seroconversion by 4 weeks [37]. The antibody response tends to be slower in older people, and significantly lower peak titers are reached after vaccination in elderly recipients [31].

#### Varicella-zoster

A study by Sonia Nader et al. compared the cell-mediated immune response to the Varicella zoster vaccine in both adults and children 6 weeks following vaccination in 2 children’s groups and 2 adult groups. Following the first dose, it was evident that there was a significantly higher response in the children’s groups than in the adult groups. CMI response was reevaluated at 1 year and was higher among the pediatric population showing persistence of T-cell proliferation at 1 year in 94% of adults in comparison with 97% of children [38].

As regards humoral immunity, the development of IgG antibodies to VZV antigen was higher 6 weeks after the first dose in children than in adults. The geometric mean titers in children in one of the children’s groups were significantly higher than one of the adults’ groups who received the same vaccine; however, in total, combined data of both children groups and both adult groups show no significant difference. IgG antibodies persisted 1 year following vaccination in all children and only 94% of adults.

#### Pertussis

A cohort study by Bette Liu et al. done on a group of 333 cases and 506 control with a mean age of 61 years reveals that vaccine effectiveness in this age group was 52%. The protection within 5 years after vaccination was described as “modest” by the authors [39].

Table 2 displays the vaccine examples.

**Table 2:** Vaccine examples showing decreased seroconversion in the elderly

Vaccine	Authors [reference number in text]	Title
Influenza	Goodwin et al. [20]	Antibody response to influenza vaccination in the elderly: a quantitative review
	Meng et al. [21]	Immunogenicity of influenza vaccine in elderly people: a systematic review and meta-analysis of randomized controlled trials, and its association with real-world effectiveness
	Zhang et al. [22]	Immunogenicity of H5N1 influenza vaccines in elderly adults: a systematic review and meta-analysis
	Yao et al. [23]	Frailty is associated with impairment of vaccine-induced antibody response and increase in post-vaccination influenza infection in community-dwelling older adults
Hepatitis B	Havlichek et al. [25]	Age-related hepatitis B seroconversion rates in health care workers
Rabies	Ceddia et al. [26]	Antibody response to rabies vaccine prepared in tissue cultures of human diploid cells and inactivated, evaluated in different classes of age
	Mastroeni et al. [27]	Immune response of the elderly to rabies vaccines
Japanese encephalitis virus	Wagner et al. [29]	Age-related differences in humoral and cellular immune responses after primary immunisation: indications for stratified vaccination schedules
Meningitis	King et al. [30]	Total and functional antibody response to a quadrivalent meningococcal polysaccharide vaccine among children
	de Costa & Martins Carlos Klein [31]	Evaluation of the protective efficacy of an antimeningococcal vaccine for serogroups B and C *Neisseria meningitidis* infections in Brazil, 1990/92
	Espín Ríos et al. [32]	Seroconversion and duration of immunity after vaccination against group C meningococcal infection in young children
Hepatitis A	Nalin et al. [33]	Worldwide experience with the CR326F-derived inactivated hepatitis A virus vaccine in pediatric and adult populations: an overview
Varicella-zoster	André et al. [34]	Clinical assessment of the safety and efficacy of an inactivated hepatitis A vaccine: rationale and summary of findings
Pertussis	Nader et al. [35]	Age-related differences in cell-mediated immunity to varicella-zoster virus among children and adults immunized with live attenuated varicella vaccine

### Does COVID-19 vaccination decrease viral shedding?

It is evident from the above that seroconversion from COVID-19 is more likely to occur in younger individuals rather than old-aged individuals. To fulfill the suggested hypothesis, that vaccination of better seroconverters can lead to a better community protection including prevention of infection of the more vulnerable groups, we need to prove that COVID-19 vaccination reduces infectivity by reducing viral load. A brief communication by Levine-Tiefenbrun and colleagues compared nasopharyngeal viral loads in vaccinated vs. unvaccinated individual 12 days after vaccination; they found that the mean viral load substantially decreased 12 days after vaccination with the first vaccine dose, coinciding with the known early onset of vaccine-mediated protection.

This could mean that changing policies of vaccine prioritization can serve as a better protection not only for vaccinated young adults but for the old aged as well.

### Ethical considerations

The idea of protecting one group through another is not new. Pregnant women, for example, consume folic acid to protect their babies, even though it could have adverse effects due to allergic reactions. Another example is the influenza vaccines, which are better in achieving immunity when targeted at children even though the elderly are getting most of the benefits [40].

The first concern is long-term safety. Kostoff et al. and Wu et al. could not guarantee the long-term safety of COVID-19 vaccines yet [41]. In order to be able to form an informed decision about child vaccination, pharmaceutical companies must continue their clinical studies to provide us with a better understanding of the long-term side effects of COVID-19 vaccines [42], especially not in subgroups such as children, pregnant women, and people with chronic illnesses; more research is needed [43].

Ensuring the safety of the vaccine for children and maintaining a risk–benefit balance is critical. However, even with limited information regarding safety in children, the WHO’s Strategic Advisory Group of Experts (SAGE) has recently announced that the Pfizer/Biotech vaccine is safe for people aged 12 years and above [44].

Other ethical problems are that children cannot choose whether they get the vaccine or not and the ability of governments to deal with vaccine refusal. There are three main justifications for vaccine refusal in the modern world:

- > The philosophical (worldview) is based on a liberal understanding of anyone’s right to self-determination of their health, body, and life. As a result, no one, including the government, can interfere with a person’s freedom to seek or refuse medical treatment.
- > The religious view advocates that those compulsory vaccinations violate the freedom of religion. Not all faiths accept vaccination. The Amish, for example, believe that vaccines are unimportant because they weaken the immune system, which serves as the body’s natural defense.
- > The medical view evaluates vaccines through side effects and possible complications. This point, especially in the context of the COVID-19 vaccines, seems justifiable, due to the lack of data on the long-term safety of the newly released vaccines.

## Conclusion

**Fig. 1:**
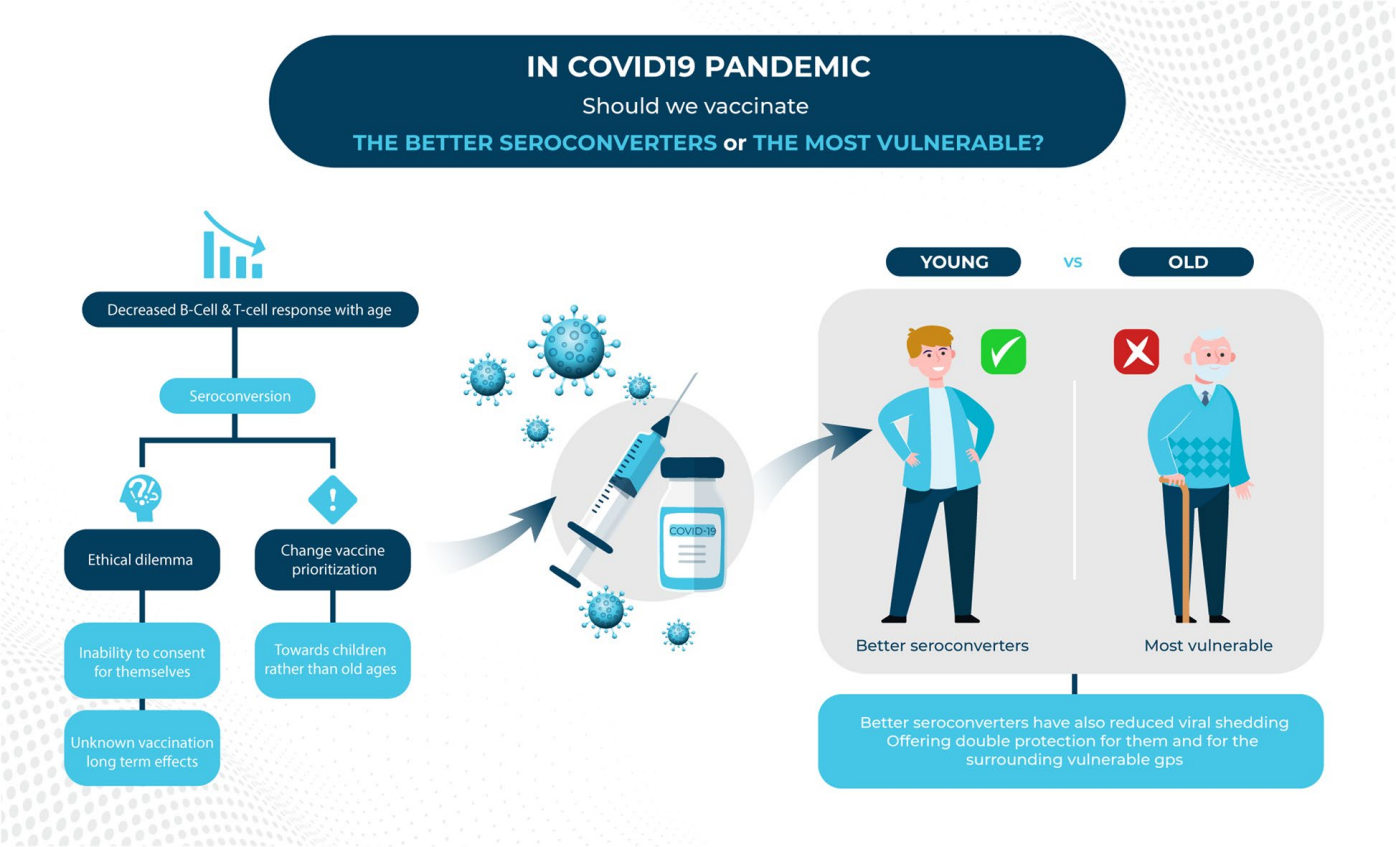
In COVID19 pandemic, should we vaccinate the better seroconverters or the most vulnerable? COVID19 coronavirus disease 2019

Children and adolescents are at higher likelihood to display a better immune response from vaccination compared with older individuals and patients with chronic diseases. Better seroconversion also reduces viral shedding. This might mean a double protection to the vaccinated individuals and to the vulnerable groups surrounding them. Adopting this policy might be a game-changer in COVID-19 vaccination prioritization policies, but some ethical dilemmas should first be discussed, the most important of which is the unknown long-term effects of the fast-tracked COVID-19 vaccines. Figure 1 summarizes the discussed aspects in the article.

## Data Availability

Not applicable

## Abbreviations

COVID-19: Coronavirus disease 2019
CSR: Class switching recombination
HDCV: Human diploid cell vaccine
Ig: Immunoglobulins
IL: Interleukin
IPV: Inactivated polio vaccine
JEV: Japanese encephalitis virus
LIF: Leukemia inhibitory factor
MAPK: Mitogen-activated protein kinases
NF: Nuclear factor
OPV: Oral polio vaccine
PPAR: Peroxisome proliferator-activated receptor
RAG: Recombinating activating genes
TGF: Transforming growth factor
TLR: Toll-like receptor
VZV: Varicella-zoster virus

## References

[1] Engla NEW, Journal ND (2021) N Engl J:589–591

[2] Wei J (2021). Antibody responses to SARS-CoV-2 vaccines in 45,965 adults from the general population of the United Kingdom. Nat Microbiol.

[3] Ciabattini A (2018). Vaccination in the elderly: the challenge of immune changes with aging. Semin Immunol.

[4] Ewer KJ (2021). T cell and antibody responses induced by a single dose of ChAdOx1 nCoV-19 (AZD1222) vaccine in a phase 1/2 clinical trial. Nat Med.

[5] Ebinger JE (2021). Antibody responses to the BNT162b2 mRNA vaccine in individuals previously infected with SARS-CoV-2. Nat Med.

[6] Deplanque D, Launay O (2021) Efficacy of COVID-19 vaccines: from clinical trials to real life. Therapies 76 10.1016/j.therap.2021.05.004 PMC8114590 34049688

[7] Ponnappan S, Ponnappan U (2011). Aging and immune function: molecular mechanisms to interventions. Antioxid Redox Signal.

[8] Stephan RP, Sanders VM, Witte PL (1996). Stage-specific alterations in murine B lymphopoiesis with age. Int Immunol.

[9] Jin R (2008). Age-related changes in BAFF and APRIL profiles and upregulation of BAFF and APRIL expression in patients with primary antibody deficieny. Int J Mol Med.

[10] Cancro MP (2009). B cells and aging: molecules and mechanisms. Trends Immunol.

[11] Riley RL, Blomberg BB, Frasca D (2005). B cells, E2A, and aging. Immunol Rev.

[12] Weksler ME (1995). Immune senescence: deficiency or dysregulation. Nutr Rev.

[13] Frasca D, Van der Put E, Riley RL, Blomberg BB (2004). Reduced Ig class switch in aged mice correlates with decreased E47 and activation-induced cytidine deaminase. J Immunol.

[14] AbdelMassih AF (2021). PPAR agonists as effective adjuvants for COVID-19 vaccines, by modifying immunogenetics: a review of literature. J Genet Eng Biotechnol.

[15] Sempowski GD (2000). Leukemia inhibitory factor, oncostatin M, IL-6, and stem cell factor mRNA expression in human thymus increases with age and is associated with thymic atrophy. J Immunol.

[16] Gruver AL, Sempowski GD (2008). Cytokines, leptin, and stress-induced thymic atrophy. J Leukoc Biol.

[17] Savino W (2006). The thymus is a common target organ in infectious diseases. PLoS Pathog.

[18] Yang H, Youm Y-H, Dixit VD (2009). Inhibition of thymic adipogenesis by caloric restriction is coupled with reduction in age-related thymic involution. J Immunol.

[19] Kumar R, Avagyan S, Snoeck HW (2010). A quantitative trait locus on chr.4 regulates thymic involution. J Gerontol Ser A Biol Sci Med Sci.

[20] Panda A (2009). Human innate immunosenescence: causes and consequences for immunity in old age. Trends Immunol.

[21] Chung HY (2009). Molecular inflammation: underpinnings of aging and age-related diseases. Ageing Res Rev.

[22] DaRoza G (2003). Stage of chronic kidney disease predicts seroconversion after hepatitis B immunization: earlier is better. Am J Kidney Dis.

[23] K, G., C, V. & L, S. (2006). Antibody response to influenza vaccination in the elderly: a quantitative review. Vaccine.

[24] Z, M. (2020). Immunogenicity of influenza vaccine in elderly people: a systematic review and meta-analysis of randomized controlled trials, and its association with real-world effectiveness. Hum Vacc Immunother.

[25] Zhang K, Wu X, Shi Y, Gou X, Huang J (2020). Immunogenicity of H5N1 influenza vaccines in elderly adults: a systematic review and meta-analysis. Hum Vacc Immunother.

[26] Yao X (2011). Frailty is associated with impairment of vaccine-induced antibody response and increase in post-vaccination influenza infection in community-dwelling older adults. Vaccine.

[27] Enani S (2018). Impact of ageing and a synbiotic on the immune response to seasonal influenza vaccination; a randomised controlled trial. Clin Nutr.

[28] Havlichek D, Rosenman K, Simms M, Guss P (1997). Age-related hepatitis B seroconversion rates in health care workers. Am J Infect Control.

[29] Antibody response to rabies vaccine prepared in tissue cultures of human diploid cells and inactivated, evaluated in different classes of age - PubMed. 6765186

[30] Mastroeni I (1994). Immune response of the elderly to rabies vaccines. Vaccine.

[31] Leder K, Weller PF, Wilson ME (2001). Travel vaccines and elderly persons: review of vaccines available in the United States. Clin Infect Dis.

[32] Wagner A (2018). Age-related differences in humoral and cellular immune responses after primary immunisation: indications for stratified vaccination schedules. Sci Rep.

[33] King WJ (1996). Total and functional antibody response to a quadrivalent meningococcal polysaccharide vaccine among children. J Pediatr.

[34] de Costa EA, Martins Carlos Klein HH (1996) Avaliação da proteção conferida pela vacina antimeningocócica BC no Estado de Santa Catarina, Brazil, 1990/92 Evaluation of the protective efficacy of an antimeningococcal vaccine for serogroups B and C Neisseria meningitidis infections in Brazil, 1990/92. 30, 460 10.1590/s0034-89101996000500009 9269096

[35] Espín Ríos I (2000). Seroconversion and duration of immunity after vaccination against group C meningococcal infection in young children. Vaccine.

[36] DR, N. (1993). Worldwide experience with the CR326F-derived inactivated hepatitis A virus vaccine in pediatric and adult populations: an overview. J Hepatol.

[37] André FE, D’Hondt E, Delem A, Safary A (1992). Clinical assessment of the safety and efficacy of an inactivated hepatitis A vaccine: rationale and summary of findings. Vaccine.

[38] Nader S, Bergen R, Sharp M, Arvin AM (1995). Age-related differences in cell-mediated immunity to varicella-zoster virus among children and adults immunized with live attenuated varicella vaccine. J Infect Dis.

[39] BC L (2020). Effectiveness of acellular pertussis vaccine in older adults: nested matched case-control study. Clin Infect Dis.

[40] Giubilini A (2021). Vaccination ethics. Br Med Bull.

[41] Kostoff RN, Briggs MB, Porter AL, Spandidos DA, Tsatsakis A (2020). Comment: COVID-19 vaccine safety. Int J Mol Med.

[42] Majeed A, Papaluca M, Molokhia M (2021). Assessing the long-term safety and efficacy of COVID-19 vaccines. J R Soc Med.

[43] Wu Q (2021). Evaluation of the safety profile of COVID-19 vaccines: a rapid review. BMC Med.

[44] Eberhardt CS, Siegrist CA (2021). Is there a role for childhood vaccination against COVID-19?. Pediatr Allergy Immunol.

